# Long term effect of phacoemulsification on intraocular pressure in patients with medically controlled primary open-angle glaucoma

**DOI:** 10.1186/s12886-019-1157-3

**Published:** 2019-07-12

**Authors:** Loic Majstruk, Benjamin Leray, Aymeric Bouillot, Sylvain Michée, Gilles Sultan, Christophe Baudouin, Antoine Labbé

**Affiliations:** 1 Department of Ophthalmology, Ambroise Paré Hospital, AP-HP, University of Versailles Saint-Quentin-en-Yvelines, Versailles, France; 2 Department of Ophthalmology III, Quinze-Vingts National Ophthalmology Hospital, IHU FOReSIGHT, 28 rue de Charenton, 75012 Paris, France; 3 INSERM U968; UPMC Univ Paris 06, UMR_S968, Institut de la Vision; CNRS, UMR 7210; CHNO des Quinze-Vingts, INSERM-DHOS CIC 503, Paris, France

## Abstract

**Background:**

The effect of cataract surgery on IOP in patients with primary open-angle glaucoma (POAG) is a subject of debate. We investigated the effect of cataract surgery by phacoemulsification on intraocular pressure (IOP) in patients with medically POAG .

**Methods:**

Seventy eyes of 40 POAG patients undergoing cataract surgery by phacoemulsification were retrospectively evaluated. All patients had their POAG medically controlled without prior glaucoma surgery. Baseline demographics and clinical characteristics were recorded. IOP and the number of glaucoma medications were evaluated before and for 1 year after cataract surgery. We analyzed IOP variations from baseline with a Student *t*-test for a paired sample. We used a Pearson correlation coefficient and linear regression to study the relation between IOP change from baseline and preoperative characteristics.

**Results:**

One year after phacoemulsification, IOP decreased by a mean 1.15 ± 3 mmHg (6.8 ± 18.1%) (*P* = 0.01) and the number of glaucoma medications remained unchanged with a difference of − 0.1 ± 0.43 (*P* = 0.09). Higher preoperative IOP was associated with a greater IOP decrease after 1 year of follow-up (*P* < 0.001). One and 7 days after cataract surgery, 12.9 and 4.2% of the eyes had IOP spikes > 30 mmHg, respectively. One year after cataract surgery, 75.7% of the POAG eyes maintained the same number of glaucoma medications while 17.1% had a decrease and 7.2% of the eyes required adding glaucoma medications.

**Conclusion:**

Cataract surgery by phacoemulsification in eyes with medically controlled POAG resulted at 1 year in a very small IOP decrease without a change in the number of glaucoma medications. A drop in IOP should not be expected after performing phacoemulsification alone in POAG patients.

## Background

The effect of cataract surgery on IOP in patients with glaucoma is a subject of debate. In patients without glaucoma, glaucoma suspects and glaucoma patients, cataract surgery by phacoemulsification has been found to lead to a small decrease in intraocular pressure (IOP) [1–7]. Shingleton et al. showed that this modest decrease in IOP is sustained until 5 years after cataract surgery [1]. Several mechanisms seem to be involved in this postoperative IOP decrease: biochemical changes with the trabecular meshwork cellular response to ultrasound [8], mechanical changes with the washout of the trabecular meshwork during phacoemulsification [9], anatomical changes with a widening of the iridocorneal angle improving trabecular meshwork access [10–12] or changes in the uveal tract increasing aqueous humor outflow [13, 14].

Lens extraction produced a significant IOP reduction in patients with primary angle-closure glaucoma (PACG) [10–12, 15]. Similarly, in pseudoexfoliation glaucoma (PXG), the washout of the trabecular meshwork during phacoemulsification seems to be responsible for the postoperative IOP decrease [9, 16–19]. Nevertheless, for POAG patients, the effect of cataract surgery on IOP and the number of medications remains unclear. In a meta-analysis for the American Academy of Ophthalmology, Chen et al. showed a modest 13% average decrease in IOP in patients with POAG after phacoemulsification alone [3]. However, studies evaluating IOP after phacoemulsification in POAG eyes showed substantial variability of results with IOP reduction ranging from − 7% [20] to − 22% [21]. Similarly, the number of glaucoma medications varied from + 7% [22] to − 59% [23] after cataract surgery. This may in be part explained by the variability of inclusion and exclusion criteria in these studies with some patients presenting with normal-tension glaucoma (NTG), uncontrolled glaucoma, or even patients with prior laser iridotomy, suggesting participation of angle closure mechanisms, and by the different study designs.

The aim of the present study was therefore to evaluate the role of cataract surgery by phacoemulsification on IOP and the number of medications in patients with medically controlled POAG. This study was conducted in a single center and the patients included had only POAG.

## Methods

In this retrospective single-center study, we evaluated patients with POAG undergoing cataract surgery by phacoemulsification between 2010 and 2015 at Ambroise Paré Hospital, AP-HP, Boulogne Billancourt, France. This study adhered to the tenets of the Declaration of Helsinki and was approved by our Institutional Review Board. A written consent was obtained from each patient.

All patients were followed in the department of ophthalmology at Ambroise Paré Hospital and had POAG diagnosed based on clinical findings (elevated IOP and optic nerve changes), visual field loss and/or retinal nerve fiber layer (RNFL) defects. Each patient had preoperative gonioscopy showing an open angle defined as gonioscopy Shaffer grade ≥ 3 in all four quadrants without peripheral anterior synechiae or heavy pigmentation, suggesting secondary or angle-closure glaucoma. At the time of surgery, the patients’ glaucoma had to be medically controlled, defined as no change in glaucoma medication regimen in the last 6 months with IOP at the target level (defined as IOP < 21 mmHg and at the individual IOP level at which physicians believe that further optic nerve progression is unlikely to occur). Patients were excluded if they had secondary glaucoma (pseudoexfoliation syndrome; pigment dispersion syndrome; uveitic, traumatic, neovascular or congenital glaucoma), or if they had had prior glaucoma surgery, laser peripheral iridotomy or laser trabeculoplasty.

The indication for cataract surgery in these patients was reduced vision attributable to a visually significant cataract. All cataract surgeries were performed with the same technique in the same department. Cataract surgery was performed by phacoemulsification with topical anesthesia, clear corneal incision (2.2 mm), Duovisc® viscoelastic (Alcon Laboratory, Fort Worth, TX, USA), standard manual anterior capsulorrhexis, a divide-and-conquer phacoemulsification technique (Infiniti®, Alcon), an intraocular lens implantation in the posterior chamber, injection of intracameral cefuroxime, and eventually stromal hydration wound closure. In the postoperative period, the patient received 1 month of a pre-established association of antibiotics and dexamethasone t.i.d., and nonsteroidal anti-inflammatory eyedrops t.i.d. Antiglaucoma medications were not stopped before surgery and were continued after surgery according to the physician’s decisions.

Baseline demographics, preoperative characteristics (gonioscopy grade, central corneal thickness, preoperative IOP, number of preoperative glaucoma medications, visual field indices, preoperative best-corrected visual acuity (BCVA)), refraction axial length, intraocular lens power and the complications of phacoemulsification were recorded. Preoperative IOP was defined as the average IOP resulting from the last three measurements prior to cataract surgery. Postoperative IOP was recorded for all follow-up visits. All IOP measurements were obtained using the same noncontact tonometer (NIDEK NT-510, Nidek CO, Gamagori, Japan), one measurement corresponding to the mean of three IOP measures. The number of preoperative and postoperative of glaucoma medications was defined as the number of active antiglaucoma molecules. Patients with POAG were evaluated before and at 1 day, 7 days, 1 month, 3 months, 6 months and 1 year after cataract surgery for IOP and the number of medications.

We analyzed IOP variations from baseline with a Student *t*-test for a paired sample. We used a Pearson correlation coefficient and linear regression to study the relation between IOP change from baseline and preoperative characteristics. Statistical analyses were made with XLSTAT software® (Addinsoft, Paris, France). A *P*-value < 0.05 was considered statistically significant.

## Results

Seventy eyes of 40 patients with POAG were included. There were 51.4% women and 48.6% men with a mean age of 77.7 ± 7.7 years. Preoperative IOP was 17 ± 2.7 mmHg with 1.5 ± 0.8 antiglaucoma medications for an average central corneal thickness of 551 ± 36.8 μm. The mean gonioscopy grading was 3.74 ± 0.5 and visual fields showed a mild to moderate glaucoma (PSD 3.3 ± 2.2 dB and MD [mean deviation] − 4 ± − 3 dB). The preoperative and demographic data are summarized in Table 1.

**Table 1:** Baseline demographics and preoperative characteristics

Characteristic	OAG (*n* = 70)
Age (y)	77.7 ± 7.7
Sex	
Male (%)	48.6
Female (%)	51.4
Gonioscopy grade (Shaffer)	3.74 ± 0.5
Central corneal thickness (υm)	551 ± 36.8
Preoperative intraocular pressure (mmHg)	17 ± 2.7
Preoperative number of glaucoma medications	1.5 ± 0.8
Visual field	
Pattern standard deviation(dB)	3.3 ± 2.2
Mean deviation (dB)	−4 ± 3
Preoperative cup/disc ratio	0.6 ± 0.2
Preoperative visual acuity (logMAR)	0.4 ± 0.7
Axial length (mm)	24 ± 2
IOL power (diopter)	19.4 ± 4.9
Posterior capsular rupture (%)	5.71
Posterior chamber IOL (%)	97.1
Sulcus implanted IOL (%)	2.9

*IOL* Intraocular lens

Values are percentages or mean ± standard deviation

**Fig. 1:**
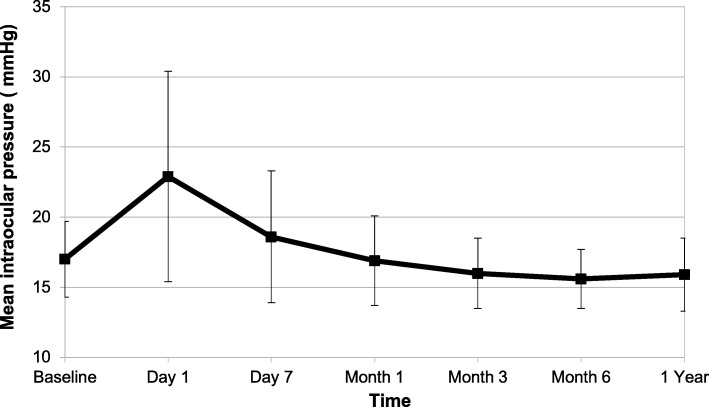
Intraocular pressure average changes after cataract surgery by phacoemulsification in patients with medically controlled primary open-angle glaucoma

After phacoemulsification, IOP increased by a mean 5.8 ± 8 mmHg at day 1 (*P* < 0.001) and a mean 1.6 ± 5 mmHg at day 7 (*P* = 0.004). Then IOP slightly decreased, with a mean variation compared to the preoperative value of 0.15 ± 4 mmHg at 1 month (*P* = 0.440), 0.96 ± 3.43 mmHg at 3 months (*P* = 0.06), 1.44 ± 2.8 mmHg at 6 months (*P* < 0.001) and by a mean 1.15 ± 3 mmHg after 1 year of follow-up (*P* = 0.01) (Fig. 1). One year after surgery, 8% of the POAG eyes had an IOP decrease > 5 mmHg; conversely 5% of the eyes had an IOP increase > 5 mmHg, and 87% had stable IOP as defined as an IOP variation < 5 mmHg. The number of patients with IOP spikes (defined as an IOP > 30 mmHg) was 12.9% at 1 day and 4.2% at 7 days and none after 1 month after surgery.

The average number of glaucoma medications was not statistically different from baseline and at each postoperative control. The average number of glaucoma medications was 1.61 ± 0.76 (*P* = 0.16) at 1 day, 1.59 ± 0.77 (*P* = 0.18) at 7 days, 1.59 ± 0.68 (*P* = 0.68) at 1 month, 1.64 ± 0.71 (*P* = 0.53) at 3 months, 1.56 ± 0.70 (*P* = 0.37) at 6 months and 1.44 ± 0.72 (*P* = 0.09) at 1 year. One year after cataract surgery, 75.7% of the POAG eyes kept the same number of glaucoma medications while 17.1% had a decrease and 7.2% of the eyes required adding glaucoma medications. No patient needed glaucoma surgery or had an additional laser treatment during follow-up.

**Fig. 2:**
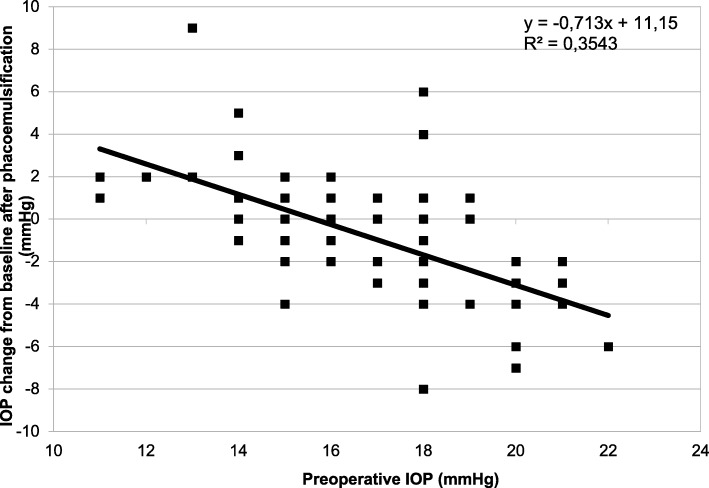
Correlation between intraocular pressure changes 1 year after cataract surgery by phacoemulsification and preoperative intraocular pressure in primary open-angle glaucoma patients

Correlation analysis revealed a statistically significant negative correlation between IOP change and preoperative average IOP (Fig. 2). This correlation was statistically significant at each postoperative visit: 1 day (r = − 0.34; *P* = 0.01), 7 days (r = − 0.37; *P* < 0.001), 1 month (r = − 0.60; *P* < 0.001), 3 months (r = − 0.68; *P* < 0.001), 6 months (r = − 0.68; *P* < 0.001) and 1 year (r = − 0.59; *P* < 0.001). No other significant correlation was detected with regard to age, gender, central corneal thickness, gonioscopy status, ophthalmic biometry, preoperative refraction, axial length, intraocular lens power, number of glaucoma medications or visual field indices (MD and PSD).

## Discussion

A recent survey of the American Glaucoma Society showed that, independently of IOP level or glaucoma stage, 44% of glaucoma surgeons performed a phacoemulsification alone in eyes with concomitant POAG and visually significant cataract, whereas 24% did a combined phacoemulsification with a trabeculectomy procedure and 22% performed a microinvasive glaucoma surgery (MIGS) associated with cataract surgery [24]. The result of the present study showed that in patients with POAG, cataract surgery by phacoemulsification can lead to a very modest (around − 1 mmHg) decrease in IOP without significant change in the number of antiglaucoma medications. Moreover, although a slight mean decrease was observed at 1 year, individual variations showed that postoperative IOP was stable (defined as an IOP variation < 5 mmHg) in the vast majority of cases (87%). These results are at the low end of the results of previous studies that showed a mean decrease in IOP of − 2.3 mmHg, ranging from − 1 to − 4 mmHg and a mean change in the number of medications of − 12% ranging from − 59 to + 7% [3]. Mierzejewski et al. [21] found the highest postoperative IOP reduction (4 mmHg) after phacoemulsification in POAG patients. Nevertheless, patients with PXG were included in their POAG group, and since no detail of gonioscopy grading was given, some eyes with a narrow angle before surgery might also explain this considerable drop in IOP following phacoemulsification. In other studies, uncontrolled POAG patients with preoperative IOP > 21 mmHg with maximum medical therapy, or NTG were also included [2, 16, 20, 22, 25, 26]. These methodological differences probably explain the high variability in the results.

In spite of this unclear long-term effect of phacoemulsification on IOP in POAG patients, some patients will experience early postoperative IOP rises. Approximately 13% of patients at 1 day and 4% at 7 days showed IOP spikes > 30 mmHg after phacoemulsification. Previous studies have also showed IOP spikes in 3–27% of POAG patients in the first postoperative days after phacoemulsification [3, 20, 27]. It is well known that POAG patients have a higher risk of IOP spikes in the early postoperative period exposing them to additional optic nerve damage on their already compromised optic disc [28, 29]. It has been reported that patients with severe glaucoma may lose fixation [30] or develop visual field progression with IOP spikes following cataract surgery [31]. Consequently, in mild to advanced glaucoma patients, IOP spikes should be avoided to protect the optic nerve from additional damage. Some patients may also undergo a long-term IOP increase. In the present study, we found that 5% of the eyes had an IOP increase > 5 mmHg and 7.2% of the eyes required adding glaucoma medications 1 year after phacoemulsification. Previous studies reported that 6–26% of glaucoma patients had long-term IOP increase with various definitions [1, 2, 15], and 4–26% of them required a glaucoma medication increase 1–5 years after phacoemulsification [2, 21]. Contrary to what it is often reported [32], these results emphasize that most patients with POAG and a widely open angle will not have a reduction in IOP after phacoemulsification and that some patients may have an immediate or a long-term IOP increase. Interestingly, in the present study we found almost the same number of POAG eyes with a postoperative IOP increase (5%) as with an IOP decrease (8%), whereas most of the eyes had a stable IOP (87%) as defined as a IOP variation < 5 mmHg.

We attempted to identify patients who experienced the greatest IOP increases or decreases following phacoemulsification. Unfortunately, we did not find a significant difference between preoperative gonioscopy grade, central corneal thickness, number of glaucoma medications, visual field indices, preoperative BCVA, refraction axial length, intraocular lens power of the POAG patients who experienced the greatest IOP changes and the other patients. The only characteristic correlated with the postoperative IOP variation was the preoperative IOP. Indeed, like Slabaugh et al. [2], we found that the postoperative IOP reduction was statistically negatively correlated with preoperative IOP in POAG patients (Fig. 2). Patients with POAG and a higher preoperative IOP were those with the highest IOP reduction after cataract surgery. The physiology of this postoperative IOP decrease in these patients remains speculative. In contrast to PCAG patients, the access for the aqueous humor to the trabecular meshwork (TM) is good (absence of iridocorneal angle narrowing or apposition), and unlike in pseudoexfoliation glaucoma there is no abnormal material to wash out during the surgical procedure. Therefore, the postoperative IOP reduction could be explained by an improved function of the trabecular meshwork rather than better access to the trabecular meshwork. A biochemical theory proposed by Wang et al. [8] in 2003 argued that a potentially IOP-lowering biochemical response may be induced on trabecular meshwork cells by ultrasound during phacoemulsification. The anatomical theory proposed by Berdal et al. [13] in 2009, after a review of the literature, suggested that the posterior lens shift after cataract surgery might relax the anterior tendons of the ciliary muscles changing the trabecular meshwork architecture and improving aqueous humor outflow. In 2010, Strenk et al. [14] analyzed magnetic resonance images of phakic and pseudophakic eyes, and found a posterior uveal shift in pseudophakic eyes. These authors also mentioned that the lens growth could displace the uveal tract anteriorly, compressing the Schlemm canal and decreasing outflow, as discussed by Berdal et al.

One hypothesis that might explain the preoperative IOP impact on the postoperative IOP decrease postulates that patients with higher preoperative IOP may have greater displacement of the uveal tract due to lens growth and consequently greater compression of the Schlemm canal and should obtain a greater benefit when the lens is extracted. Another explanation and a major limitation in evaluating the effect of phacoemulsification on IOP is the statistical regression to the mean effect. This is the tendency of a variable that is distinct from the norm to return to normal after repeated measures due to random fluctuations. Patients with higher preoperative IOP also have a higher probability of having a greater postoperative IOP reduction after repeating the test. Regression to the mean usually occurs in nonrandomized studies [33, 34], which is the case of the vast majority of the studies evaluating the effect of phacoemulsification on IOP in POAG. Nevertheless, since phacoemulsification is clearly not a treatment for POAG, it is difficult to conduct a randomized study on that subject. Further randomized studies with large samples would be necessary to identify the predictive factors of postoperative IOP variations after cataract surgery in POAG patients.

Phacoemulsification alone in patients with mild to moderate controlled POAG seems to lead to a modest, unpredictable IOP reduction and does not seem to induce changes in the number of glaucoma medications. Moreover, it can lead to an immediate postoperative IOP rise and a long-term IOP increase.

## Conclusions

Cataract surgery in POAG patients is a frequent clinical situation and many studies have shown that with modern phacoemulsification glaucoma patients can expect excellent visual outcome with no differences compared to patients without glaucoma. Despite some limitations, this study showed that for patients with medically controlled mild or moderate POAG, cataract surgery by phacoemulsification resulted in a clinically nonsignificant decrease of IOP and no change in the number of glaucoma medications after 1 year of follow-up. In such patients cataract surgery alone should not be considered an efficient IOP-lowering strategy and other options should be considered for patients with more severe disease. Careful determination of the type of glaucoma seems mandatory so as to define the most appropriate therapeutic proposal, especially if glaucoma is not properly controlled. In POAG patients, an additional procedure should therefore be considered, since the effect of standalone cataract surgery will not be very beneficial. Moreover, postoperative monitoring after cataract surgery by phacoemulsification should be carried out carefully because some patients may present IOP spikes that should be treated rapidly.

## Data Availability

The datasets used and/or analysed during the current study are available from the corresponding author on reasonable request.

## Abbreviations

BCVA: Best corrected visual acuity
IOP: Intraocular pressure
MD: Mean deviation
MIGS: Micro invasive glaucoma surgery
NTG: Normal tension glaucoma
PACG: Primary angle-closure glaucoma
POAG: Primary open-angle glaucoma
PSD: Pattern standard deviation
PXG: Pseudo exfoliation glaucoma
RNFL: Retinal nerve fiber layer
TM: Trabecular meshwork
